# The lipoxygenase gene family: a genomic fossil of shared polyploidy between *Glycine max *and *Medicago truncatula*

**DOI:** 10.1186/1471-2229-8-133

**Published:** 2008-12-23

**Authors:** Jin Hee Shin, Kyujung Van, Dong Hyun Kim, Kyung Do Kim, Young Eun Jang, Beom-Soon Choi, Moon Young Kim, Suk-Ha Lee

**Affiliations:** 1 Department of Plant Science, Seoul National University, Seoul 151-921, Korea; 2 National Instrumentation Center for Environmental Management, Seoul National University, Seoul 151-921, Korea; 3 Research Institute for Agriculture and Life Sciences, Seoul National University, Seoul 151-921, Korea; 4 Plant Genomic and Breeding Research Institute, Seoul National University, Seoul, 151-921, Korea

## Abstract

**Background:**

Soybean lipoxygenases (*Lxs*) play important roles in plant resistance and in conferring the distinct bean flavor. *Lxs *comprise a multi-gene family that includes *GmLx1*, *GmLx2 *and *GmLx3*, and many of these genes have been characterized. We were interested in investigating the relationship between the soybean lipoxygenase isozymes from an evolutionary perspective, since soybean has undergone two rounds of polyploidy. Here we report the tetrad genome structure of soybean *Lx *regions produced by ancient and recent polyploidy. Also, comparative genomics with *Medicago truncatula *was performed to estimate *Lxs *in the common ancestor of soybean and *Medicago*.

**Results:**

Two *Lx *regions in *Medicago truncatula *showing synteny with soybean were analyzed. Differential evolutionary rates between soybean and *Medicago *were observed and the median Ks values of Mt-Mt, Gm-Mt, and Gm-Gm paralogs were determined to be 0.75, 0.62, and 0.46, respectively. Thus the comparison of Gm-Mt paralogs (Ks = 0.62) and Gm-Mt orthologs (Ks = 0.45) supports the ancient duplication of *Lx *regions in the common ancestor prior to the *Medicago*-*Glycine *split. After speciation, no *Lx *regions generated by another polyploidy were identified in *Medicago*. Instead tandem duplication of *Lx *genes was observed. On the other hand, a lineage-specific duplication occurred in soybean resulting in two pairs of *Lx *regions. Each pair of soybean regions was co-orthologous to one *Lx *region in *Medicago*. A total of 34 *Lx *genes (15 *MtLxs *and 19 *GmLxs) *were divided into two groups by phylogenetic analysis. Our study shows that the *Lx *gene family evolved from two distinct *Lx *genes in the most recent common ancestor.

**Conclusion:**

This study analyzed two pairs of *Lx *regions generated by two rounds of polyploidy in soybean. Each pair of soybean homeologous regions is co-orthologous to one region of *Medicago*, demonstrating the quartet structure of the soybean genome. Differential evolutionary rates between soybean and *Medicago *were observed; thus optimized rates of Ks per year should be applied for accurate estimation of coalescence times to each case of comparison: soybean-soybean, soybean-*Medicago*, or *Medicago*-*Medicago*. In conclusion, the soybean *Lx *gene family expanded by ancient polyploidy prior to taxon divergence, followed by a soybean- specific duplication and tandem duplications, respectively.

## Background

Lipoxygenases (LOXs) have been intensively studied for the past century and have been reported in yeast, algae, fungi, animals, and plants [1]. In higher plants, LOXs are almost ubiquitous and involved in various physiological processes. Importantly, the oxidized products by these enzymes are involved in the traumatic acid and jasmonic acid (JA) pathways, which confer various biotic and abiotic resistance traits to plants [2-4]. Soybean lipoxygenases have received significant attention, since the oxidized compounds cause soy products to have an unpleasant flavor [1,5,6]. Many lipoxygenase isozymes have been isolated and are well characterized, but these studies were mainly focused on LOX 1, 2 and 3, the lipoxygenases preferentially expressed in seeds. Moreover, an analysis of soybean mutant lines lacking the isozymes has shown that the *Lx1 *and *Lx2 *loci are tightly linked, while the *Lx3 *locus is independent of the other two loci [7,8]. Additionally, several vegetative *Lx *genes, such as *Lx4*, *Lx5*, *Lx6*, *Lx7*, and *Lx8*, have been detected and characterized [9,10].

A number of *Lx *loci have been identified in the fully sequenced *Arabidopsis thaliana *and almost completed *Medicago *genomes. This suggests that there are more *Lx *genes not yet reported in the soybean genome, and that the number of soybean *Lxs *would outnumber those in *Arabidopsis *and *Medicago*. The expansion of a gene family is related to gene duplication events, such as whole genome duplication, tandem duplication, and transposition [11]. An investigation of the *Lx *gene family expansion is also of interest in the context of soybean genome evolution, since soybean is known to have undergone two rounds of polyploidy events by analyses of ESTs [12,13]. The duplicated soybean genome has been investigated by RFLP mapping and more than two regions have been detected by RFLP probes [14]. Furthermore, analyses of homeologous BAC clones, anchored by *FAD2 *and *HCBT *genes, revealed highly conserved regions produced by a recent duplication 14.5 million years ago (MYA) [15,16]. The presence of duplicated soybean chromosomal regions was substantiated by analyzing seventeen homeologous BACs [17]. These studies also showed the genome rearrangement of homeologous regions after whole genome duplication.

Genome duplication events are common in most crop plants [18]. Even model plants, such as *Arabidopsis *and *Medicago*, have undergone at least one round of genome doubling [12,19]. A comparative genomic approach using *Medicago *has provided insights into complex legume genomes, which cannot be satisfactorily studied provided by the model plant *A. thaliana *[20]. The divergence of soybean and *Medicago *from a common ancestor which experienced genome duplication was estimated at 50 MYA [14]. *Medicago *and soybean, two closely related legume plants, have had two bursts of gene duplication, but it is not clear whether they had a shared polyploidy event before taxon divergence. To clarify this issue, a phylogenetic analysis of gene families was performed, and a hypothesis of shared polyploidy prevailed over the alternative hypothesis of taxon divergence prior to duplication [21]. Later, the early duplication of soybean before the *Medicago*-soybean split was supported by the whole genome duplication which predated speciation between *Medicago *and *Lotus japonicus *[22].

With regard to genome conservation in legumes, many studies have shown broad-scale conservation of legume genomes and gene order [23,24]. Not only among soybean, *Medicago *and *Arabidopsis *[24] but microsynteny was also observed among three genomes [25]. Mudge *et al*. (2005) [26] identified very high synteny between 3 Mb of soybean DNA sequences and 2 *Medicago *chromosomes. A recent study also demonstrated a network of synteny within conserved regions among *Arabidopsis*, *Medicago *and soybean [27].

In this study the evolutionary expansion of the soybean *Lx *gene family was demonstrated to occur by two rounds of polyploidy and the evolutionary relationships of nineteen *Lx *genes in four homeologous chromosomal regions were explored. Moreover, the differential rates of evolution in orthologous and paralogous regions of the *Lx *gene regions between soybean and *Medicago *reflect the history of the paleopolyploid soybean genome.

## Results

### Assembly and Mapping of Soybean BACs and Scaffolds

**Figure 1:**
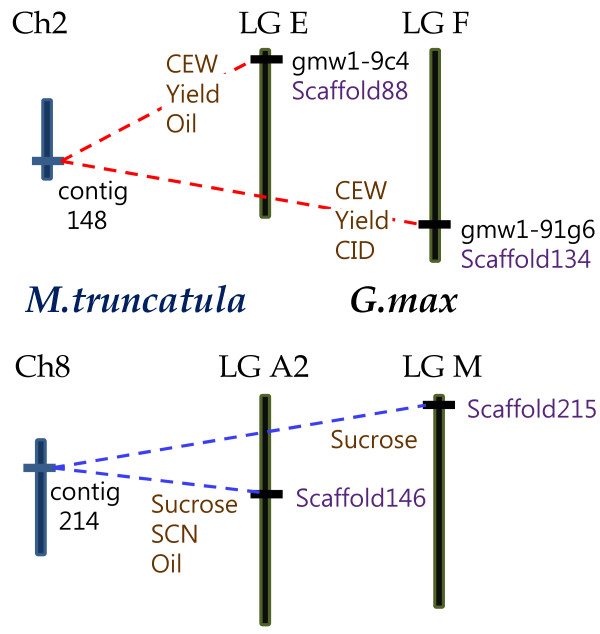
**Comparative map of six *Lx *regions from soybean and *Medicago***. *In silico *genetic mapping with SSR markers placed the soybean *Lx *regions into four different linkage groups (LGs). Two BAC clones, gmw1-9c4 and gmw1-91g6, were selected containing *Lx1*, *Lx2 *and *Lx3*, and these genes were embedded in Scaffolds 88 and 134. Two blocks showing synteny with selected BACs were detected in *Medicago *and contig 148 on *Medicago *chromosome 2 was more similar to the BACs. Blast search with the sequence of contig 214 on *Medicago *chromosome 8 resulted in two more soybean *Lx *scaffolds. Soybean QTLs for each *Lx *region are denoted as brown characters and some of them are conserved among the four regions.

**Figure 2:**
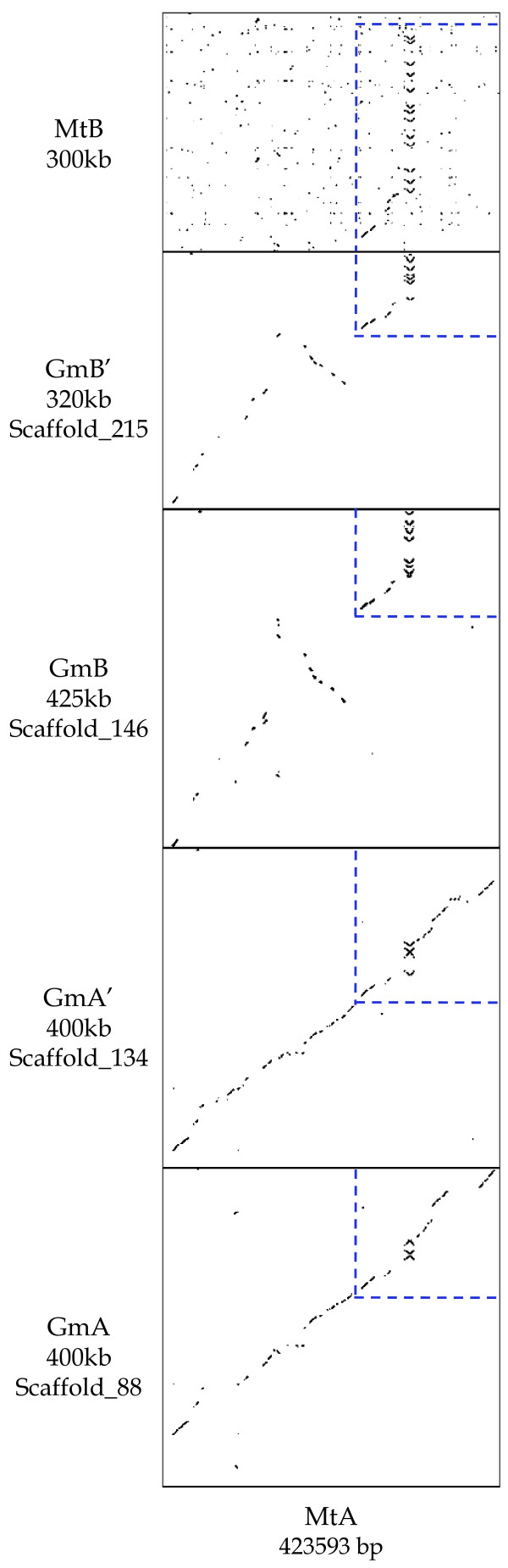
**Dot plot alignments of six *Lx *regions between soybean and *Medicago***. MtA shows a high level of similarity with GmA and GmA'. Both GmB and GmB' have an inversion block, and their sequences end with repetitive lipoxygenases like MtB. Common sequences among the six regions are highlighted with blue dotted lines.

PCR-based screens of the gmw1 BAC library identified a total of six BAC clones: gmw1-45b2 and gmw1-91g6 for both *Lx1 *and *Lx2; *gmw1-6b18, gmw1-9c4, gmw1-22a20, and gmw1-22f19 for *Lx3*. In other words, *Lx1 *and *Lx2 *are located on the same BACs, but *Lx3 *is on different BACs, which is in accordance with many previous reports [8,28]. The six BAC clones were sequenced using 454 sequencing technology and the average read length was 250 bp. The number of contigs varied from 1 to 25 and the largest assembled contig was 36 kb (see Additional file 1). Two BAC clones, gmw1-22a20 and gmw1-22f19, were fully sequenced. The remaining gaps were closed by hybridization assemblies, adding ABI-Sanger sequences amplified across the gaps [29]. Here, we mainly used gmw1-9c4 containing *Lx3 *and gmw1-91g6 containing *Lx1 *and *Lx2 *for further analysis (Figure 1). More than ten scaffolds containing lipoxygenase genes were identified from the 7× whole genome sequencing (WGS) assembly from early 2008  and selected scaffolds showing synteny with BAC clones were analyzed for further study. Sequences of gmw1-9c4 and gmw1-91g6 were embedded in Scaffold 88 and Scaffold 134, respectively. Also, the sequences of Scaffold 146 and Scaffold 215 were highly identical to each other and showed colinearity with those BAC clones (Figures 1 and 2). Scaffold 88, Scaffold 134, Scaffold 146, and Scaffold 215 were named GmA, GmA', GmB, and GmB', respectively. A total of 13 soybean *Lx *genes were searched on NCBI and nine of them were included in GmA, GmA', GmB, and GmB'. Two of the soybean *Lx *genes did not have proper scaffolds with high scores, and the scaffold containing GmLOX9 did not show any synteny except for the GmLOX9 gene itself (see Additional file 2).

Genetic mapping was achieved by identification of lipoxygenase genes and simple sequence repeat (SSR) markers placed on the composite map . Previously, *GmLx1 *and *GmLx2 *on Scaffold 134 were mapped to linkage group (LG) F and *GmLx3 *on Scaffold 88 was mapped to LG E [7,28,30]. By *in silico *mapping based on sequence, the four scaffolds were placed on four different LGs: Scaffold 88 was anchored by Satt575, Satt213, Sat_112, and Satt411 on LG E; Scaffold 134 contained Sat_090, Satt656, and Sat_417 on LG F; Scaffold 146 had Sat_115, Sat_199, Sat_129, Sat_233, and Satt089 on LG A2; Scaffold 215 was mapped to LG M by Sat_389, Satt404 and Sat_391.

Numerous QTLs have been related to these four *Lx *regions in soybean and some of them have been associated with more than one region: corn earworm resistance (CEW) and yield QTLs on part of LG E and LG F [31-34]; sucrose content QTLs on LG A2 and LG M [35], oil QTLs on LG E and LG A2 [36,37]. These mutually conserved QTLs indicate that specific genes associated with CEW, yield, sucrose, and oil have been retained across homeologous genomic regions after genome duplication (Figure 1). Additionally, the carbon isotope discrimination (CID) on LG F and soybean cyst nematode resistance (SCN) on LG A2 have been reported [32,38].

### Comparison of *Lx *Regions in *G. max *and *M. truncatula*

Two *Lx *regions colinear to these two soybean BACs were detected on *Medicago *chromosomes 2 and 8 in *Medicago *pseudomolecule 2.0  and named MtA and MtB, respectively (Figure 2). MtA consists of five BAC clones: AC148918, AC137554, AC146308, AC136955 and AC155896. MtB is comprised of four BAC clones: AC149580, AC140032, AC149638 and AC174341. A dot-plot analysis of the six *Lx *regions between soybean and *Medicago *revealed that all showed synteny with some genome rearrangement by insertion, deletion, and tandem duplication. MtA shared most of the genes with the two soybean BACs; however, Mt8 contig 214 showed synteny with only short regions of the both ends of the soybean BACs, with tandem duplicated *Lxs *being observed instead. Also, a search in the *Medicago *database  identified 32 *Lx *gene loci. Only 15 *Lxs *in these two regions were further analyzed because the remaining loci did not show any synteny with soybean *Lx *regions.

**Figure 3:**
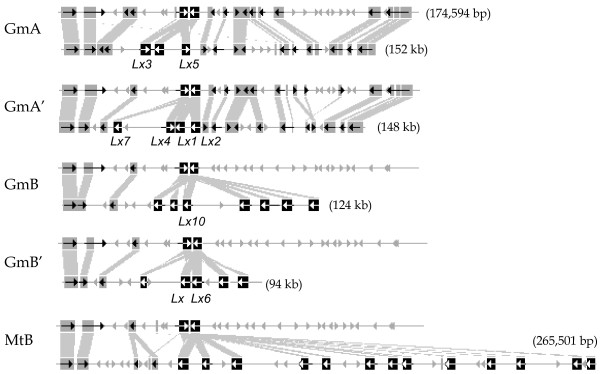
**Diagrammatic representation of gene conservation between the six *Lx *regions by BLASTZ**. The sequence highlighted with blue dotted lines in Figure 2 was analyzed in detail with gene prediction. The length and orientation of predicted genes are represented as arrows, and homologous sequences are connected with grey boxes. Each lipoxygenase is depicted as a black box and white arrow, and soybean *Lx *genes registered in GenBank are denoted with their gene names. A total of 34 *Lx *loci, 15 from *Medicago *and 19 from soybean, were detected.

Detailed gene structure and comparisons of the six *Lx *regions are shown by blue dotted lines (Figure 2) and BLASTZ (Figure 3). The Ks values between homologous genes were calculated (see Additional file 3). Full annotation of the genes is available in Additional file 4. A total of 15 pairs of combinations between the six regions were compared based on their Ks values (Table 1). By comparing the median Ks values of common genes among the six regions, differential evolutionary rates between *Medicago *and soybean were observed. The median Ks value between MtA and MtB was 0.75, which was close to the *Medicago *older peak estimated by other analyses [12,13,22]. The median Ks value between Gm-Gm paralogs was similar to previous reports [12,13]. However, the median Ks value between Gm-Mt paralogs was smaller than Mt-Mt paralogs, but larger than Gm-Gm paralogs (Tables 1 and 2). The median Ks value of Gm-Mt orthologs was almost the same as that of Gm-Gm paralogs. The median Ks value of GmA-GmA' and GmB-GmB' were 0.11 and 0.10, respectively, suggesting they were produced by a recent polyploidy in soybean like the event defining the *FAD2 *gene family and *HCBT *gene regions [15,16].

**Table 1:** Median Ks values for combinations of pairs between six *Lx *regions from *Medicago *and soybean

Combinations of pairs	Median Ks
Ancient polyploidy	
Mt-Mt paralog	
MtA-MtB	0.75
Gm-Mt paralog	
MtA-GmB	0.58
MtA-GmB'	0.60
MtB-GmA	0.64
MtB-GmA'	0.67
Gm-Gm paralog	
GmA-GmB	0.46
GmA-GmB'	0.45
GmA'-GmB	0.48
GmA'-GmB'	0.46
Taxon divergence	
Gm-Mt ortholog	
MtA-GmA	0.40
MtA-GmA'	0.41
MtB-GmB	0.49
MtB-GmB'	0.50
Recent polyploidy	
Gm-Gm paralog	
GmA-GmA'	0.11
GmB-GmB'	0.10

**Table 2:** Ks estimations of ancient polyploidy and taxon divergence

Materials	Mt-Mt paralogs^a^	Gm-Mt paralogs^a^	Gm-Gm paralogs^a^	Gm-Mt orthologs^b^	References
ESTs	0.65–0.70	-	0.45–0.50	0.40–0.50	Blanc and Wolfe, 2004
ESTs	0.71	-	0.54	-	Schlueter *et al*., 2004
39 Gene families	-	-	0.57± 0.05	0.57± 0.02	Pfeil *et al*., 2005
Lipoxygenases	0.75	0.62^c^	0.46	0.45^d^	This study

^a^The median Ks value between paralogs represents polyploidy events.

^b^The median Ks value between orthologs denotes taxon divergence between soybean and *Medicago*.

^c, d ^The median Ks values between Gm-Mt paralogs and Gm-Mt orthologs are compared to rule out skewing estimates caused by differential substitution rates. Thus, the ancient polyploidy predates the taxon divergence.

The gene density of the six *Lx *regions was similar: one gene per 7.06 kb in MtA; one gene per 8.11 kb in MtB; one gene per 7.27 kb in GmA; one gene per 7.55 kb in GmA'; one gene per 7.59 kb in GmB; one gene per 7.62 kb in GmB'. The density of these regions in *Medicago *was not significantly different from that of the homologous regions in soybean, consistent with previous reports of one gene per 6 kb or 5.8–6.7 kb [16,26,39]. The average GC content was approximately the same among those regions: 32.68% in MtA; 32.52% in MtB; 32.14% in GmA; 32.05% in GmA'; 31.96% in GmB; 31.17% in GmB'. Among the six *Lx *regions in this study, GmA and GmA' were more similar to MtA, whereas GmB and GmB' were closer to MtB (Figs. 2, 3).

### Phylogenetic Analysis of *Lx *Genes in Soybean and *Medicago*

**Figure 4:**
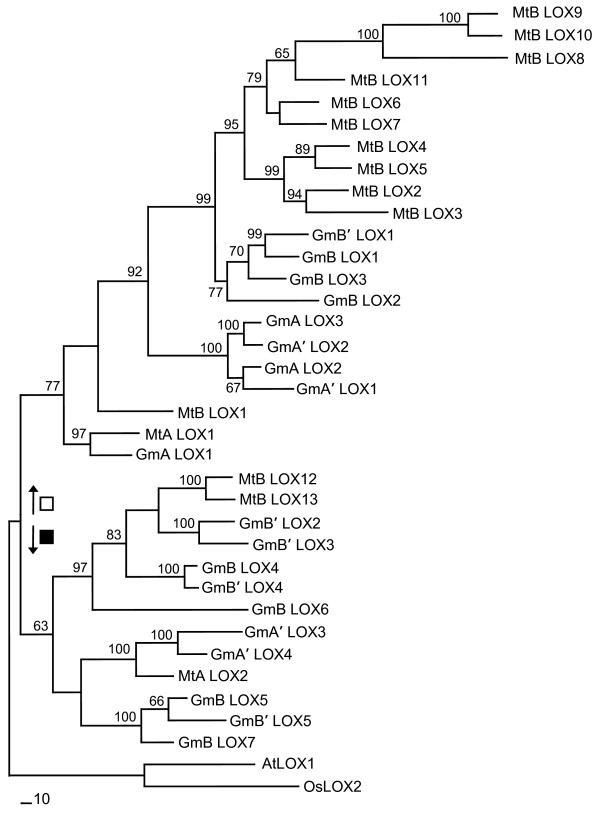
**Phylogenetic analysis of 34 *Lx *proteins**. A parsimony tree was generated using bootstrap analysis with 1,000 replicates and branch swapping. Bootstrap values larger than 50 are denoted on each branch. The tree was rooted using *Arabidopsis *LOX1 (AtLOX1) and rice LOX1(OsLOX1). Soybean and *Medicago Lxs *in homologous regions represented as black and white squares fall into two major clades. For convenience, each *Lx *gene is named according to its species, chromosomal region and physical order instead of its GeneID. The *Lxs *in GmA diverged earlier from *Lxs *in GmB and MtB and later *Lxs *in GmB and MtB are separated, suggesting duplication before taxon divergence.

A total of 34 *Lxs *were detected from the six homologous *Lx *regions: 2 in MtA; 13 in MtB; 3 in GmA; 4 in GmA'; 7 in GmB; 5 in GmB' (Figure 3). For convenience, each *Lx *gene was named according to its species, chromosomal region, and physical order. Thus, their designated names are different from their GeneIDs in GenBank. Because the *Lx *gene structures were very similar, their evolutionary relationships were uncovered by calculating their Ks values. The Ks values among ten *Lxs *(from *MtB_Lx2 *to *MtB_Lx11*) ranged from 0.3440 to 0.6393, indicating extensive tandem duplication of *Lx *genes after whole genome duplication in *Medicago*. Phylogenetic analysis using parsimony of 34 *Lx *genes in the six regions classified these 34 *Lx *genes into two clades denoted as black and white squares (Figure 4). The grouping of *Lx *genes showed that GmB *Lxs *were more similar to MtB *Lxs *than to GmA or GmA' *Lxs*. In other words, the divergence time between GmA and GmB was earlier than the time of speciation between the two species. After taxon divergence, GmA and GmB regions were duplicated resulting in GmA, GmA', GmB, and GmB'. In *Medicago*, the tandem duplication of *Lx *genes was observed instead of another polyploidy.

## Discussion

### Ancient polyploidy in the *Lx *regions of common ancestor

Previously, it had not been clear whether soybean and *Medicago *shared a polyploidy event because the old peak of paralog Ks in *Medicago *did not overlap with that of the soybean [12,13]. To explain the gap between the soybean and *Medicago *paralog Ks peaks, Blanc and Wolfe hypothesized that the soybean lineage split from one of the allopolyploid genomes of *Medicago *[12]. Later, an analysis of gene families provided a framework of shared polyploidy prior to taxon divergence [21]. A total of 56% of gene families also supported a shared soybean-*Medicago *duplication before the split, whereas the remaining gene families supported alternative hypotheses, including taxon divergence prior to the ancient polyploidy [40]. In addition, a lower synonymous substitution rate in soybean was suggested to explain the difference between *Medicago *and soybean Ks value peaks [13,40].

Our data corroborates the hypothesis that the two peaks of median Ks values in soybean and *Medicago *actually represent the same event but show differential synonymous substitution rates. While orthologs refer to homologous genes that have been generated by speciation, paralogs are homologous genes generated via duplication [41]. The median Ks values of Mt-Mt paralogs, Gm-Mt paralogs, and Gm-Gm paralogs revealed the differential evolutionary rates between the two species (Table 1). The median Ks value of Mt-Mt paralogs (Ks = 0.75) is greater than that of Gm-Gm paralogs (Ks = 0.46), while the Gm-Mt value is intermediate. Thus, to decide the chronological order of duplication and taxon divergence without bias produced by differential evolutionary rates, it is absolutely crucial to compare the values within the same category. A comparison of Gm-Mt paralogs (Ks = 0.62) and Gm-Mt orthologs (Ks = 0.45) indicates that ancient duplication occurred prior to speciation (Table 2). In conclusion, the Mt-Mt, Gm-Mt and Gm-Gm paralogs actually represent the same duplication event, although their absolute values look different.

Recently, a large-scale duplication between *Medicago *and *L. japonicus *was proven to have occurred before speciation [22]. The Ks distribution of ancient duplication between *Medicago *and *Lotus *was not significantly different, even though *Medicago *had a narrower peak and *Lotus *showed a broader peak. The median Ks value of older polyploidy in *Medicago *and *Lotus *had been estimated to be 0.7 to 0.9 [12,13,22]. In our study, the Ks value of older polyploidy in soybean was much smaller, consistent with previous studies (Table 2) [12,13,21]. Thus, optimized rates of Ks per year should be applied for balanced estimation of coalescence times to each case of comparison: soybean-soybean, soybean-*Medicago *or *Medicago*-*Medicago*.

Most crop legumes belong to the Hologalegina and phaseoloid-millettioid clades [42]. The earlier duplication between *Medicago *and *Lotus *is the duplication event in the common ancestor of the Hologalegina clade, which includes *Medicago*, *Lotus*, and *Pisum*. Soybean belongs to the phaseoloid-millettioid clade, which contains *Glycine*, *Phaseolus*, and *Vigna*. Taken together, our data support an ancient duplication event in the common ancestor of the Hologalegina and phaseoloid-millettioid clades.

### Evolutionary change of soybean and *Medicago *after speciation

It has been suggested that the younger peak in *Medicago *did not correspond to another polyploidy but a series of tandem duplications because the peak was too broad [21,40]. Also, there was no clear Ks peak suggesting large scale duplication after the *Medicago*-*Lotus *split [22]. In this study, only two *Medicago Lx *regions produced by ancient polyploidy were detected, and no chromosomal region generated by recent duplication was identified. Instead, ten occurrences of extensive single gene duplication were observed in one *Medicago Lx *region. The colinearity between MtA and MtB was not high except for repetitive *Lxs *and a few flanking genes (Figure 2). It is thought that these duplicated regions were differentiated by a diploidization process.

A total of four soybean chromosomal regions were anchored by three to seven *Lx *genes. Among the four *Lx *regions, the level of similarity and sequence conservation was high between regions produced by the recent duplication (Figures. 2, 3). These two pairs of *Lx *regions were generated by two rounds of polyploidy in soybean. With respect to the conservation level of sequence and structure, both inter- and intra-pairs showed synteny (e.g. GmA-GmB, GmA-GmB', GmA'-GmB, and GmA'-GmB'). The level of diploidization in soybean *Lx *regions generated by ancient polyploidy was lower than that of *Medicago*. Moreover, the conserved QTLs among the four regions- sucrose, oil, yield, and corn earworm resistance- support their duplicated origin (Figure 1).

Until now, sequence-based analyses of the soybean genome have been focused on regions produced by recent polyploidy [15-17,43]. The comparative genomics approach used in this paper furthered our understanding of the soybean genome and allowed us to speculate on chromosomal regions produced by both recent and ancient duplication events. Furthermore, each pair of *Lx *regions in soybean was close to an *Lx *region in *Medicago*. Co-orthologs refer to genes generated by a lineage-specific duplication [41]. Thus, GmA/GmA' and GmB/GmB' are the co-orthologous chromosomal regions to MtA and MtB, respectively (Figure 1). In this case, it is difficult to conclude whether *Medicago *and soybean, are allo- or autopolyploids. But it is clear that soybean and *Medicago *share both of the genome, rejecting the hypothesis of *Medicago *allopolyploid history after the *Medicago*-soybean split.

### Expansion and functional divergence of the *Lx *gene family

**Figure 5:**
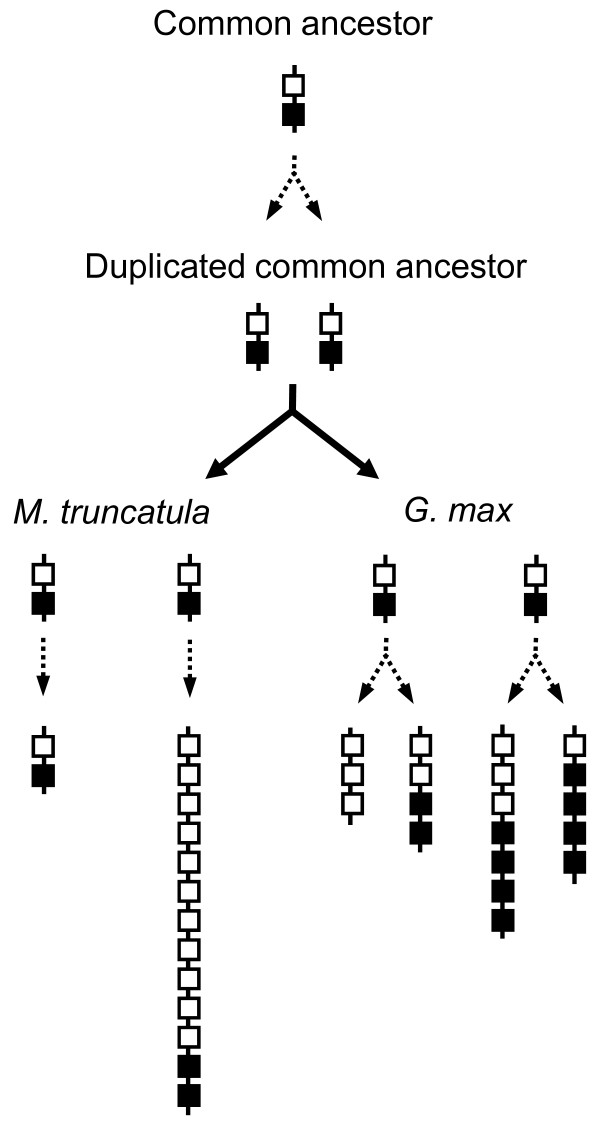
**Expansion of the *Lx *gene family in soybean and *Medicago *homologous regions in relation to the evolutionary events in the six regions**. No direct evidence of a recent polyploidy event in *Medicago *was detected; instead, tandem duplication of *Lxs *was observed in MtB. In soybean, two pairs of *Lx *regions generated by two rounds of polyploidy were identified and analyzed. Each pair of soybean regions is co-orthologous to the region in *Medicago*, suggesting co-orthologous regions produced by lineage-specific duplication in soybean.

Phylogenetic analysis divided 34 *Lx *genes identified in six *Lx *regions from soybean and *Medicago *into two clades (Figure 4). We expect two distinct *Lx *genes in the most recent common legume ancestor. This parsimony tree showed that the *Lx *genes in the GmB region were closer to the *Lx *genes in MtB than those of GmA, suggesting duplication prior to taxon divergence. After the split, tandem duplication of *Lx *genes occurred in MtB, whereas the soybean *Lx *genes duplicated to GmA *Lx*/GmA' *Lx *and GmB *Lx*/GmB' *Lx*. This evolution of the *Lx *gene family in the homologous regions is diagrammatically represented in Figure 5.

Duplicated genes have been reported to undergo non-functionalization, neo-functionalization, or sub-functionalization [44]. Among the 19 *Lx *genes in the four soybean regions, nine were previously characterized and confirmed functional (see Additional file 2). In addition, the duplicated lipoxygenase genes had different activities at different pH values and different substrate specificities, suggesting differential functional specificities among lipoxygenase isoforms [9,45]. Moreover, the patterns of cellular and subcellular localization in pod walls were distinct among the isoforms, indicating independent functions [46]. Specialized isoforms are expected to improve the plant's flexibility to various environmental conditions.

Retention of multiple copies of *Lxs *in soybean, *Medicago*, and their common ancestor are reasonable from an evolutionary perspective because lipoxygenases confer various biotic and abiotic resistance traits to plants. Plant lipoxygenases have been reported to conferred resistance to stresses such as herbivores and wounding [47,48]. Also, clusters of genes related to resistance and disease response have been reported in soybean [15,49]. In grape (*Vitis vinifera*), the gene family encoding the grapevine phytoalexin is comprised of 43 genes, 20 of which were previously shown to be expressed [50]. Numerous *Lx *genes will increase protein or mRNA dosage, leading to resistance in plants. The beneficial effects of increased dosage of genes involved in defense or resistance has been reported in various studies: resistance to glyphosate in plants, protection against heavy metals in hamsters, and decreased susceptibility to HIV infection in humans [51-53]. This mechanism of gene family expansion and functional divergence of duplicated genes may also be relevant to understanding the evolution of other gene families.

A systematic approach is required for crop improvement and modification because most crops have more than one gene copy in their genomes. It is absolutely essential to investigate the number of loci of a particular gene of interest in the breeding of polyploid crops. Further understanding and insights into the paleopolyploid crop genome will lead to more efficient crop improvement and molecular breeding.

## Conclusion

In this study, multiple *Lx *genes anchored in four soybean regions and two *Medicago *regions were analyzed at the sequence level. Differential evolutionary rates between soybean and *Medicago *were revealed among the six regions, with *Medicago *showing a greater synomymous substitution rate than soybean. This fact suggests that an optimized coalescence estimation is needed for each comparison: Gm-Gm, Gm-Mt or Mt-Mt. The four soybean *Lx *regions are comprised of two pairs of recently duplicated regions, and each pair is co-orthologous to one region in *Medicago*. These results support an ancient polyploidy in the common ancestor of soybean and *Medicago*, which preceded separation of the Hologalegina and phaseoloid-millettioid clades. Based on the tetrad soybean genome structure, four copies of duplicated genes or four homeologous regions in soybean are theoretically expected. Phylogenetic analysis showed that the *Lx *gene family basically expanded by whole genome duplication. Moreover, *Lx *genes underwent extensive tandem gene duplication.

## Methods

### Lipoxygenase BAC selection and mining of soybean super contigs

Three specific PCR primers were designed to select BAC clones that contained the target genes, *Lx1*, *Lx2 *and *Lx3*, based on GenBank acc. numbers J02795, J03211, and U50081, respectively. The primer sequences were: *Lx1 *forward, 5'-TTA ATG CTT TCT TGG GCC CTA-3' and *Lx1 *reverse, 5'-CGC TCT CCC GTT CCA TTT CC-3'; *Lx2 *forward, 5'-GCT ATA AAT CAC GTT TCG TTA C-3' and *Lx2 *reverse, 5'-TAT GCC CTC CTC CTC TGT TC-3'; *Lx3 *forward, 5'-GTAGTGTTGGTGGGTTGCAAAGATG-3' and *Lx3 *reverse, 5'-GCA AAC AAA GTG GAT GCT TCC ATG-3'.

A pilot experiment was performed with *G. max *cultivar Williams 82, prior to BAC selection to optimize PCR conditions using a PTC-225-DNA gradient cycler from MJ research (Watertown, MA., USA). Williams 82 was used as a positive control during the selection procedure. The amplification reaction was 11 μl in volume and contained 100 ng of Williams 82 genomic DNA, 15 pmol of each forward and reverse primer, 10.0 mM of dNTP mix, 1 μl of 10× buffer, 6.9 μl of dd-H_2_O, and 0.2 unit of *Taq *DNA polymerase (Vivagen, Sungnam, Korea). The PCR conditions were 94°C for 2 min, 35 cycles of 94°C for 30 sec, annealing temperature for 30 sec, 72°C for 30 sec, and a final extension of 2 min at 72°C.

The Williams 82 *G. max *BAC clone library [54] was PCR-screened using the same conditions as described above for the genomic DNA of Williams 82. The final PCR screen was conducted with 0.2 μl of the candidate BACs as a template from a working copy of the library.

Soybean super contigs (scaffolds) were identified by BLAST search with lipoxygenase genes against the soybean genome sequence produced by the Soybean Genome Project, DOE Joint Genome Institute .

### *In silico *mapping of BACs and super contigs

Genetic markers for *Lx1*, *Lx2 *and *Lx3 *were defined on the consensus soybean genetic map (December, 2006; ) and the sequences of the accessions from which the SNP-containing sequence tagged site was developed were compared with BAC clone sequences using BLAST2 . Simple sequence repeat (SSR) markers in BAC clones and scaffolds were identified by BLAST search against genome survey sequence (GSS) records restricted to soybean SSR-containing clones . Thus, the genetic map positions of the selected BAC clones and scaffolds were determined by the loci of lipoxygenase and SSR markers.

### BAC sequencing and assembly

Six BACs, gmw1-45b2 (EU028318), gmw1-91g6 (EU028319), gmw1-6b18 (EU028314), gmw1-9c4 (EU028315), gmw1-22a20 (EU028316) and gmw1-22f19 (EU028317), were sequenced using Genome Sequencer (GS)-FLX. Sequence data were assembled using Phred, Phrap, and Consed to diminish the number of contigs. The remaining gaps were closed by hybrid assembly [29], adding ABI-Sanger sequences from the end of the contigs.

### Sequence analysis and annotation

Repetitive sequences were screened using RepeatMasker .

Gene prediction of soybean and *Medicago *sequences was performed using FgeneSH on an *Arabidopsis *matrix, because the results were better suited for BLASTZ results than that of the *Medicago *matrix . Each predicted gene was annotated by BLASTP searches against UniProt. Syntenic regions in *M. truncatula *were detected using the BLASTN program with nucleotide collection restricted to *M. truncatula*. These syntenic regions were compared with Pipmaker [55], BLASTZ program, and visualized using SynBrowse  and GBrowse .

### Nucleotide substitution rates, dating of duplication events, and phylogenetic analysis

The Ks values between putative homologues were calculated using the PAML package [56]. Sequences of lipoxygenases in the six *Lx *regions of soybean and *Medicago *were compiled and aligned using ClustalX and sequence overhang at the 5'- and 3'-end of alignments were removed. A parsimony tree was generated using bootstrap analysis with 1,000 replicates and branch swapping in PAUP* 4.0 [57] and rooted with *Arabidopsis *and rice as out-groups.

## Authors' contributions

JHS designed this study, selected BACs, produced the phylogenetic tree and analyzed the sequences. KV estimated Ks values and helped to design and draft the manuscript. DHK identified and sequenced BAC selections and annotated BACs. KDK mined syntenic regions in *Medicago truncatula*, performed comparative genomics as well as Ks value estimation and mapped BACs and scaffolds *in silico*. BSC sequenced BACs and assembled their sequences. YEJ helped to design primers and select BAC clones. MYK helped to draft the manuscript. SHL helped to design this study as well as draft the manuscript. All authors have read and approved the final manuscript.

## Supplementary Material

Additional File 1 **Assembly statistics of six BAC clones from GS-FLX.** This data provided show the assembly statistics of six BAC clones from GS-FLX and the remaining gaps were closed by hybridization assemblies, adding ABI-Sanger sequences amplified across the gaps. Click here for file

Additional File 2 **List of GenBank GeneIDs corresponding to soybean *Lx *genes with their phylogenetic relationships.** A total of 13 soybean *Lx *genes were searched on NCBI and nine of them were included in GmA, GmA', GmB, and GmB' Click here for file

Additional File 3 **Pairwise comparisons of Ks values between homologous genes.** These Ks values of common genes among the six homologous regions show differential evolutionary rates between *Medicago *and soybean. Click here for file

Additional File 4 **Descriptions of predicted genes based on UniRef results within the six *Lx *regions.** This table provides descriptions of predicted genes and *Lx *genes are highlighted with green color. Click here for file
