# Supplementary material for: The lipoxygenase gene family: a genomic fossil of shared polyploidy between Glycine max and Medicago truncatula
Source: BMC Plant Biol. 2008 Dec 23;8:133. doi: 10.1186/1471-2229-8-133 (PMC2644698; doi:10.1186/1471-2229-8-133)
Supplement: Additional File 1 — Assembly statistics of six BAC clones from GS-FLX. This data provided show the assembly statistics of six BAC clones from GS-FLX and the remaining gaps were closed by hybridization assemblies, adding ABI-Sanger sequences amplified across the gaps. [file 1471-2229-8-133-S1.doc]

**Additional file 1.** Assembly statistics of six BAC clones from GS-FLX.

| BAC | Number  of reads^a^ | Contigs | Total  lentgh^b^ (bp) | Average contig length (bp) | Largest contig (bp) |
| --- | --- | --- | --- | --- | --- |
| gmw1-45b2 | 9,654 | 25 | 142,680 | 5,707 | 35,726 |
| gmw1-91g6 | 5,749 | 15 | 116,356 | 7,757 | 19,573 |
| gmw1-6b18 | 6,228 | 18 | 159,654 | 8,869 | 27,116 |
| gmw1-9c4 | 5,192 | 20 | 130,993 | 6,549 | 15,468 |
| gmw1-22a20 | 6,554 | 1 | 36,048 | 36,048 | 36,048 |
| gmw1-22f19 | 7,771 | 1 | 35,942 | 35,942 | 35,942 |

^a^ Number of reads assembled

^b^ Total length in assembly
