# Supplementary material for: The lipoxygenase gene family: a genomic fossil of shared polyploidy between Glycine max and Medicago truncatula
Source: BMC Plant Biol. 2008 Dec 23;8:133. doi: 10.1186/1471-2229-8-133 (PMC2644698; doi:10.1186/1471-2229-8-133)

**Additional file 2.** Lists of GenBank GeneIDs corresponding to soybean *Lx* genes with their phylogenetic relationships.


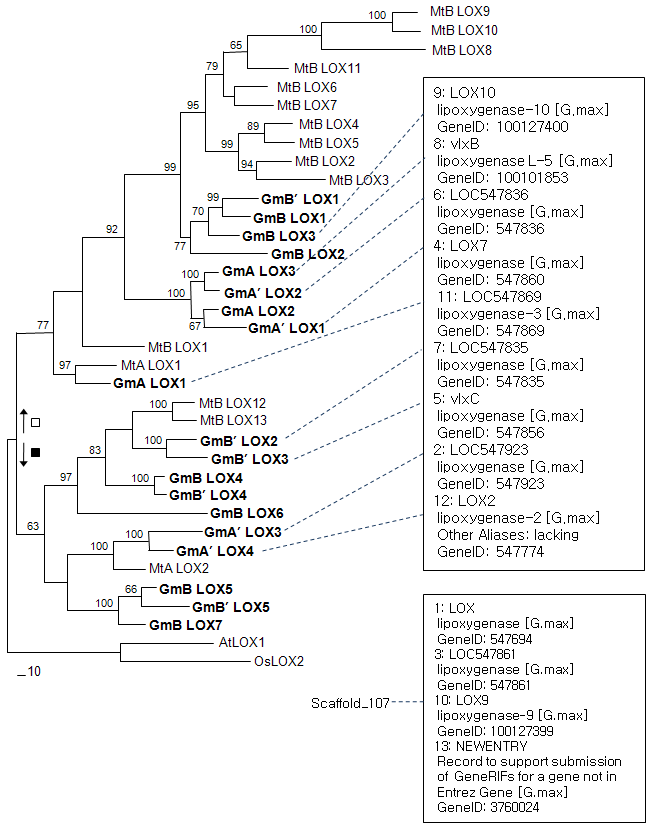

Supplement: Additional File 2 — List of GenBank GeneIDs corresponding to soybean Lx genes with their phylogenetic relationships. A total of 13 soybean Lx genes were searched on NCBI and nine of them were included in GmA, GmA', GmB, and GmB' [file 1471-2229-8-133-S2.doc]
