# Supplementary material for: The lipoxygenase gene family: a genomic fossil of shared polyploidy between Glycine max and Medicago truncatula
Source: BMC Plant Biol. 2008 Dec 23;8:133. doi: 10.1186/1471-2229-8-133 (PMC2644698; doi:10.1186/1471-2229-8-133)
Supplement: Additional File 3 — Pairwise comparisons of Ks values between homologous genes. These Ks values of common genes among the six homologous regions show differential evolutionary rates between Medicago and soybean. [file 1471-2229-8-133-S3.doc]

**Additional file 3.** Pairwise comparisons of Ks values between homologous genes.

|  | SEQ1 | SEQ2 | Ka | Ks | Ka/Ks | PROT_% | CDNA_% |
| --- | --- | --- | --- | --- | --- | --- | --- |
| Mt-Mt paralog | |  |  |  |  |  |  |
|  | 1.MtA-MtB |  |  |  |  |  |  |
|  | MtA_1 | MtB_1 | 0.0949 | 0.6456 | 0.1471 | 83.14 | 83.42 |
|  | MtA_2 | MtB_2 | 0.0917 | 0.7759 | 0.1181 | 83.69 | 83.19 |
|  | MtA_5 | MtB_8 | 0.2207 | 0.7556 | 0.2921 | 68.52 | 75.1 |
|  | MtA_5 | MtB_11 | 0.0215 | 0.7506 | 0.0286 | 94.19 | 86.44 |
|  |  |  | Median | 0.7531 |  |  |  |
| Gm-Mt paralog | |  |  |  |  |  |  |
|  | 2. MtA-GmB |  |  |  |  |  |  |
|  | MtA_1 | GmB_1 | 0.0793 | 0.5821 | 0.1363 | 85.12 | 85.04 |
|  | MtA_2 | GmB_2 | 0.0960 | 0.6501 | 0.1476 | 83.69 | 84.04 |
|  | MtA_5 | GmB_4 | 0.0212 | 0.4733 | 0.0448 | 94.19 | 88.86 |
|  |  |  | Median | 0.5821 |  |  |  |
|  | 3. MtA-GmB' |  |  |  |  |  |  |
|  | MtA_1 | GmB'_1 | 0.0809 | 0.5967 | 0.1356 | 84.87 | 84.79 |
|  | MtA_2 | GmB'_2 | 0.0899 | 0.6217 | 0.1447 | 84.32 | 84.6 |
|  | MtA_5 | GmB'_4 | 0.0213 | 0.4576 | 0.0465 | 94.19 | 89.1 |
|  |  |  | Median | 0.5967 |  |  |  |
|  | 4. MtB-GmA |  |  |  |  |  |  |
|  | GmA_1 | MtB_1 | 0.0913 | 0.6267 | 0.1457 | 83.87 | 83.78 |
|  | GmA_2 | MtB_2 | 0.0832 | 0.5737 | 0.1450 | 85.53 | 85.53 |
|  | GmA_4 | MtB_8 | 0.2204 | 0.6929 | 0.3181 | 68.83 | 75.62 |
|  | GmA_4 | MtB_11 | 0.0168 | 0.6446 | 0.0260 | 95.88 | 88.22 |
|  |  |  | Median | 0.6357 |  |  |  |
|  | 5. MtB-GmA' |  |  |  |  |  |  |
|  | MtB_1 | GmA'_1 | 0.0908 | 0.6545 | 0.1387 | 84.03 | 83.57 |
|  | MtB_2 | GmA'_2 | 0.0803 | 0.5830 | 0.1378 | 84.38 | 85.16 |
|  | MtB_8 | GmA'_4 | 0.2199 | 0.7526 | 0.29227 | 68.83 | 75.1 |
|  | MtB_11 | GmA'_4 | 0.0170 | 0.6903 | 0.0246 | 95.88 | 87.73 |
|  |  |  | Median | 0.6724 |  |  |  |
|  | Median Ks value of all Gm-Mt paralog | | | 0.6242 |  |  |  |
| Gm-Gm paralog | |  |  |  |  |  |  |
|  | 6. GmA-GmB | |  |  |  |  |  |
|  | GmA_1 | GmB_1 | 0.0719 | 0.5132 | 0.1401 | 85.39 | 85.69 |
|  | GmA_2 | GmB_2 | 0.0858 | 0.4606 | 0.1862 | 84.89 | 86.6 |
|  | GmA_4 | GmB_4 | 0.0097 | 0.4603 | 0.0211 | 97.34 | 90.4 |
|  |  |  | Median | 0.4606 |  |  |  |
|  | 7. GmA-GmB' | |  |  |  |  |  |
|  | GmA_1 | GmB'_1 | 0.0738 | 0.5009 | 0.1473 | 85.92 | 86.2 |
|  | GmA_2 | GmB'_2 | 0.0844 | 0.4471 | 0.1888 | 84.68 | 86.88 |
|  | GmA_4 | GmB'_4 | 0.0097 | 0.4455 | 0.0219 | 97.34 | 90.64 |
|  |  |  | Median | 0.4471 |  |  |  |
|  | 8. GmA'-GmB | |  |  |  |  |  |
|  | GmA'_1 | GmB_1 | 0.0654 | 0.5358 | 0.1220 | 86.69 | 85.78 |
|  | GmA'_2 | GmB_2 | 0.0833 | 0.4673 | 0.1783 | 84.15 | 86.32 |
|  | GmA'_4 | GmB_4 | 0.0098 | 0.4827 | 0.0202 | 97.34 | 90.07 |
|  |  |  | Median | 0.4827 |  |  |  |
|  | 9. GmA'-GmB' | |  |  |  |  |  |
|  | GmA'_1 | GmB'_1 | 0.0671 | 0.5263 | 0.1275 | 87.32 | 86.33 |
|  | GmA'_2 | GmB'_2 | 0.0802 | 0.4573 | 0.1753 | 84.38 | 86.64 |
|  | GmA'_4 | GmB'_4 | 0.0098 | 0.4559 | 0.0215 | 97.34 | 90.8 |
|  |  |  | Median | 0.4573 |  |  |  |
|  | Median Ks value of all Gm-Gm paralog | | | 0.4640 |  |  |  |

**Additional file 3.** Continued

|  | SEQ1 | SEQ2 | Ka | Ks | Ka/Ks | PROT_% | CDNA_% |
| --- | --- | --- | --- | --- | --- | --- | --- |
| Gm-Mt ortholog | |  |  |  |  |  |  |
|  | 10. MtA-GmA | |  |  |  |  |  |
|  | MtA_1 | GmA_1 | 0.0560 | 0.3899 | 0.1437 | 88.63 | 88.98 |
|  | MtA_2 | GmA_2 | 0.0614 | 0.4091 | 0.1501 | 84.74 | 86.08 |
|  | MtA_4 | GmA_3 | 0.1183 | 0.4056 | 0.2916 | 78.35 | 85.57 |
|  | MtA_5 | GmA_4 | 0.0212 | 0.4035 | 0.0524 | 94.19 | 90.15 |
|  |  |  | Median | 0.4046 |  |  |  |
|  | 11. MtA-GmA' | |  |  |  |  |  |
|  | MtA_1 | GmA'_1 | 0.0547 | 0.3996 | 0.1368 | 89.36 | 88.87 |
|  | MtA_2 | GmA'_2 | 0.0642 | 0.4347 | 0.1476 | 87.21 | 87.91 |
|  | MtA_5 | GmA'_4 | 0.0212 | 0.4126 | 0.0515 | 94.19 | 89.59 |
|  |  |  | Median | 0.4126 |  |  |  |
|  | 12. MtB-GmB | |  |  |  |  |  |
|  | MtB_1 | GmB_1 | 0.0802 | 0.4758 | 0.1685 | 85.77 | 86.15 |
|  | MtB_2 | GmB_2 | 0.0760 | 0.4540 | 0.1674 | 86.62 | 87.47 |
|  | MtB_8 | GmB_4 | 0.2154 | 0.5470 | 0.3938 | 68.83 | 77.26 |
|  | MtB_11 | GmB_4 | 0.0184 | 0.5087 | 0.0361 | 95.4 | 89.75 |
|  |  |  | Median | 0.4923 |  |  |  |
|  | 13. MtB-GmB' | |  |  |  |  |  |
|  | MtB_1 | GmB'_1 | 0.0813 | 0.4680 | 0.1738 | 85.53 | 86.15 |
|  | MtB_2 | GmB'_2 | 0.0735 | 0.4454 | 0.1650 | 87.05 | 87.47 |
|  | MtB_8 | GmB'_4 | 0.2230 | 0.5963 | 0.3740 | 68.52 | 76.44 |
|  | MtB_11 | GmB'_4 | 0.0157 | 0.5314 | 0.0296 | 95.88 | 89.67 |
|  |  |  | Median | 0.4997 |  |  |  |
|  | Median Ks value of all Gm-Mt ortholog | | | 0.4454 |  |  |  |
| Gm-Gm paralog | |  |  |  |  |  |  |
|  | 14. GmA-GmA' | |  |  |  |  |  |
|  | GmA_1 | GmA'_1 | 0.0159 | 0.1055 | 0.1507 | 96.62 | 96.54 |
|  | GmA_2 | GmA'_2 | 0.0250 | 0.0766 | 0.3269 | 94.87 | 96.43 |
|  | GmA_4 | GmA'_4 | 0.0001 | 0.1300 | 0.0010 | 100 | 96.85 |
|  |  |  | Median | 0.1055 |  |  |  |
|  | 15. GmB-GmB' | |  |  |  |  |  |
|  | GmB_1 | GmB'_1 | 0.0127 | 0.1000 | 0.1270 | 97.3 | 96.84 |
|  | GmB_2 | GmB'_2 | 0.0170 | 0.0874 | 0.1948 | 96.2 | 96.77 |
|  | GmB_4 | GmB'_4 | 0.0029 | 0.1263 | 0.0228 | 99.52 | 96.77 |
|  |  |  | Median | 0.1000 |  |  |  |
