# Supplementary material for: The lipoxygenase gene family: a genomic fossil of shared polyploidy between Glycine max and Medicago truncatula
Source: BMC Plant Biol. 2008 Dec 23;8:133. doi: 10.1186/1471-2229-8-133 (PMC2644698; doi:10.1186/1471-2229-8-133)
Supplement: Additional File 4 — Descriptions of predicted genes based on UniRef results within the six Lx regions. This table provides descriptions of predicted genes and Lx genes are highlighted with green color. [file 1471-2229-8-133-S4.doc]

**Additional file 4.** Descriptions of predicted genes based on UniRef results with the six *Lx* regions.

| MtA | GmA | GmA' | Descriptions |
| --- | --- | --- | --- |
| MtA_1 | GmA_1 | GmA'_1 | Putative phospholipid-transporting  ATPase 4 - *Arabidopsis thaliana* |
| MtA_2 | GmA_2 | GmA'_2 | Putative uncharacterized protein  - *Vitis vinifera* |
| MtA_3 |  |  | Putative polyprotein - *Oryza sativa* |
| MtA_4 | GmA_3 |  | - |
|  |  | GmA'_3 | - |
| MtA_5 | GmA_4 | GmA'_4 | Eukaryotic initiation factor 4A-11 |
|  |  |  | - *Nicotiana tabacum* |
| MtA_6 |  |  | MYB transcription factor MYB107 - *G. max* |
| MtA_7 |  |  | - |
| MtA_8 |  |  | Putative uncharacterized protein - *O. sativa* |
|  | GmA_5 |  | - |
| MtA_9 (MtA_Lx1) |  |  | Seed lipoxygenase-3 - *Glycine max* |
| MtA_10 (MtA_Lx2) |  |  | Lipoxygenase - *G. max* |
|  | GmA_6 (GmA_Lx1) |  | Seed lipoxygenase-3 - *G. max* |
|  | GmA_7 (GmA_Lx2) |  | Lipoxygenase - *G. max* |
|  | GmA_8 (GmA_Lx3) |  | Lipoxygenase-5 - *G. max* |
|  |  | GmA'_5(GmA'_Lx1) | Lipoxygenase - *G. max* |
|  |  | GmA'_6 | Lipoxygenase - *G. max* |
|  |  | GmA'_7 (GmA'_Lx2) | Lipoxygenase-4 - *G. max* |
|  |  | GmA'_8 (GmA'_Lx3) | Seed lipoxygenase-1 - *G. max* |
|  |  | GmA'_9 (GmA'_Lx4) | Seed lipoxygenase-2 - *G. max* |
| MtA_11 | GmA_9 | GmA'_10 | - |
| MtA_12 |  |  | - |
|  | GmA_10 |  | - |
| MtA_13 | GmA_11 | GmA'_11 | - |
| MtA_14 | GmA_12 | GmA'_12 | - |
| MtA_15 | GmA_13 | GmA'_13 | Threonine synthase, chloroplast precursor |
|  |  |  | - *Solanum tuberosum* |
|  |  | GmA'_14 | - |
|  |  | GmA'_15 | PHD3 - *G. max* |
|  | GmA_14 |  | - |
|  | GmA_15 |  | - |
|  | GmA_16 |  | F3N23.2 protein - *A. thaliana* |
| MtA_16 | GmA_17 | GmA'_16 | PHD3 - *M. truncatula* |
| MtA_17 | GmA_18 |  | - |
|  |  | GmA'_17 | - |
| MtA_18 | GmA_19 | GmA'_18 | - |
|  |  | GmA'_19 | Endochitinase PR4 precursor |
|  |  |  | - *Phaseolus vulgaris* |
| MtA_19 |  | GmA'_20 | Class IV chitinase precursor - *M. truncatula* |
| MtA_20 |  |  | Class IV chitinase precursor - *M. truncatula* |
| MtA_21 |  |  | - |
| MtA_22 |  |  | - |
| MtA_23 |  |  | - |
| MtA_24 |  |  | - |
| MtA_25 |  |  | - |
| MtA_26 |  |  | - |
|  | GmA_20 |  | - |
| MtA_27 | GmA_21 | GmA'_21 | Expressed protein - *A. thaliana* |
| MtA_28 |  |  | - |
|  | GmA_22 | GmA'_22 | - |
| MtA_29 | GmA_23 | GmA'_23 | NIN-like protein 2 - *Lotus japonicus* |

**Additional file 4. Continued**

| MtB | GmB | GmB' | Descriptions |
| --- | --- | --- | --- |
| MtB_1 | GmB_1 | GmB'_1 | Putative phospholipid-transporting  ATPase 4 - *A. thaliana* |
| MtB_2 | GmB_2 | GmB'_2 | Putative uncharacterized protein  - *V. vinifera* |
| MtB_3 |  |  | - |
| MtB_4 |  |  | - |
| MtB_5 |  |  | - |
| MtB_6 |  |  | - |
| MtB_7 |  |  | - |
|  | GmB_3 | GmB'_3 | - |
| MtB_8 | GmB_4 | GmB'_4 | Eukaryotic initiation factor 4A-9  - *N. tabacum* |
| MtB_9 |  |  | - |
| MtB_10 |  |  | RNA-directed DNA polymerase , putative |
|  |  |  | - *M. truncatula* |
| MtB_11 |  |  | - |
|  | GmB_5 | GmB'_5 | Glycylpeptide N-tetradecanoyltransferase 1 |
|  |  |  | - *A. thaliana* |
|  | GmB_6 | GmB'_6 | MYB transcription factor MYB91 - *G. max* |
|  | GmB_7 |  | Lipoxygenase-10 - *G. max* |
| MtB_12 |  |  | MYB transcription factor MYB91 - *G. max* |
| MtB_13 (MtB_Lx1) |  |  | Lipoxygenase - *Vicia faba* |
| MtB_14 (MtB_Lx2) |  |  | Lipoxygenase - *Lens culinaris* |
| MtB_15 |  |  | - |
| MtB_16 (MtB_Lx3) |  |  | Lipoxygenase - *Pisum sativum* |
| MtB_17 |  |  | - |
| MtB_18 |  |  | - |
| MtB_19 (MtB_Lx4) |  |  | Lipoxygenase - *P. sativum* |
| MtB_20 (MtB_Lx5) |  |  | Lipoxygenase - *Caragana jubata* |
| MtB_21 |  |  | Lipoxygenase-10 - *G. max* |
| MtB_22 |  |  | - |
| MtB_23 (MtB_Lx6) |  |  | Lipoxygenase - *L. culinaris* |
| MtB_24 (MtB_Lx7) |  |  | Lipoxygenase - *L. culinaris* |
| MtB_25 (MtB_Lx8) |  |  | Lipoxygenase - *P. sativum* |
| MtB_26 |  |  | - |
| MtB_27 (MtB_Lx9) |  |  | Lipoxygenase loxN2 - *P. sativum* |
| MtB_28 |  |  | - |
| MtB_29 |  |  | - |
| MtB_30 (MtB_Lx10) |  |  | Lipoxygenase loxN2 - *P. sativum* |
| MtB_31 (MtB_Lx11) |  |  | Lipoxygenase - *P. sativum* |
| MtB_32 |  |  | - |
| MtB_33 (MtB_Lx12) |  |  | Lipoxygenase - *P. sativum* |
| MtB_34 (MtB_Lx13) |  |  | Lipoxygenase - *P. sativum* |
|  | GmB_8 (GmB_Lx1) |  | Lipoxygenase-10 - *G. max* |
|  | GmB_9 (GmB_Lx2) |  | Lipoxygenase-10 - *G. max* |
|  | GmB_10 (GmB_Lx3) |  | Lipoxygenase-10 - *G. max* |
|  | GmB_11 |  | - |
|  | GmB_12 (GmB_Lx4) |  | Seed lipoxygenase - *G. max* |
|  | GmB_13 (GmB_Lx5) |  | Seed lipoxygenase-2 - *G. max* |
|  | GmB_14 (GmB_Lx6) |  | Seed lipoxygenase - *G. max* |
|  | GmB_15 (GmB_Lx7) |  | Seed lipoxygenase-2 - G. max |
|  |  | GmB'_7 (GmB'_Lx1) | Lipoxygenase - *C. jubata* |
|  |  | GmB'_8 | - |
|  |  | GmB'_9 (GmB'_Lx2) | Seed lipoxygenase - *G. max* |
|  |  | GmB'_10 (GmB'_Lx3) | Lipoxygenase - *P. sativum* |
|  |  | GmB'_11 | - |
|  |  | GmB'_12 (GmB'_Lx4) | Seed lipoxygenase - *G. max* |
|  |  | GmB'_13 (GmB'_Lx5) | Lipoxygenase - *P. sativum* |
